# Potential anticancer properties and mechanisms of thymoquinone in osteosarcoma and bone metastasis

**DOI:** 10.1186/s11658-022-00320-0

**Published:** 2022-03-02

**Authors:** Mina Homayoonfal, Zatollah Asemi, Bahman Yousefi

**Affiliations:** 1 Research Center for Biochemistry and Nutrition in Metabolic Diseases, Institute for Basic Sciences, Kashan University of Medical Sciences, Kashan, Islamic Republic of Iran; 2 Molecular Medicine Research Center, Tabriz University of Medical Sciences, Tabriz, Iran; 3 Department of Biochemistry, Faculty of Medicine, Tabriz University of Medical Sciences, Tabriz, Iran

**Keywords:** Osteosarcoma, Bone metastasis, Thymoquinone, Signaling pathway, Apoptosis, Angiogenesis, Chemotherapy resistance

## Abstract

Despite great advances, therapeutic approaches of osteosarcoma, the most prevalent class of preliminary pediatric bone tumors, as well as bone-related malignancies, continue to demonstrate insufficient adequacy. In recent years, a growing trend toward applying natural bioactive compounds, particularly phytochemicals, as novel agents for cancer treatment has been observed. Bioactive phytochemicals exert their anticancer features through two main ways: they induce cytotoxic effects against cancerous cells without having any detrimental impact on normal cell macromolecules such as DNA and enzymes, while at the same time combating the oncogenic signaling axis activated in tumor cells. Thymoquinone (TQ), the most abundant bioactive compound of *Nigella sativa*, has received considerable attention in cancer treatment owing to its distinctive properties, including apoptosis induction, cell cycle arrest, angiogenesis and metastasis inhibition, and reactive oxygen species (ROS) generation, along with inducing immune system responses and reducing side effects of traditional chemotherapeutic drugs. The present review is focused on the characteristics and mechanisms by which TQ exerts its cytotoxic effects on bone malignancies.

## Introduction

Osteosarcoma (OS) is known as the most aggressive cancerous bone disease and occurs mostly in children and adolescents [1]. Osteosarcoma or osteogenic sarcoma, Ewing tumors, and chondrosarcoma are the most common types of preliminary bone tumors [2]. The worldwide incidence of OS, known as the most prevalent principal malignant tumor of bone, has been reported to be about one to three cases per million per year [3, 4]. Based on age, the diagram of OS occurrence is presented as a bimodal distribution curve in which two distinct peaks are recognizable; the first peak can be seen in children and adolescents, while the second peak emerges in people over the age of 50 years [5]. OS accounts for approximately 60% of the widespread histological subclasses of pediatric bone sarcoma [6]. OS is categorized as a mesenchymal neoplasm of malignancy in which the cancer cells directly generate imperfect osteoid tissue such as chondroblastic, conventional, high-grade surface, low-grade central, secondary, periosteal, parosteal, or small-cell varieties. It should be noted that producing these tumours most often arise in the long bones from osteoid-producing neoplastic cells adjacent to the growth plates, occurring less commonly in other nonlong bones and the axial skeleton [7, 8]. OS shows a strong tendency to emerge in bone cells with an explosive growth rate [9].

Environmental and epidemiological parameters as well as genetic disruption play a role in the etiology of OS. Age, alkylating agents, bone turnover, chromosomal abnormalities such as hereditary retinoblastoma, sex, height, ionizing radiation, and Paget’s disease are important risk factors associated with OS progression [10]. OS can occur in all bones, although it primarily affects the long bone metaphysis. The detection of malignant osteoblasts and their outcomes, such as osteoid osteoma, leads to the clinical diagnosis of OS [11]. The typical treatment for patients suffering from OS is the combination of neoadjuvant multiagent chemotherapy and surgical resection, which may increase the survival rate of patients; however, OS recurrence and metastasis increase the mortality rate [12], with the survival rate decreasing to less than 30% for patients with tumor metastasis to the lung [13]. Furthermore, application of typical chemotherapeutic agents such as high dosage of 5-fluorouracil, adriamycin, cisplatin, methotrexate, doxorubicin, and/or etoposide as well as ifosfamide may result in both acute and long-term toxicity [14]. Moreover, patients with metastatic osteosarcoma show inadequate response to currently used chemotherapeutic agents [15]. Hence, there is a pressing need to discover more efficient chemotherapeutic agents with fewer side effects to eradicate primary OS and suppress metastasis to enhance long-term survival rates.

Recently, convincing evidence has demonstrated that components isolated from natural plant products have a wide range of biological effects, including antioxidant, anti-inflammatory, and anticancer properties [16–18]. Thymoquinone (C_10_H_12_O_2_; TQ) is a volatile oil ingredient derived from *Nigella sativa* Linn. seeds. *Nigella sativa* is generally identified as black cumin seeds of the Ranunculaceae botanical family. Black cumin seeds have been applied as a natural product for the treatment of atopic dermatitis (eczema) and bronchial asthma in the traditional medicine of Middle Eastern for more than 2000 years [19]. Furthermore, several in vivo and in vitro observations have revealed the antineoplastic activities of TQ against a broad variety of liquid and solid tumors, with few side effects [20]. TQ has anticancer effects against various types of cancer cells, including colon, lung, myeloblastic leukemia, ovarian, pancreas, and osteosarcoma [21]. TQ exerts its antitumor features by affecting different cellular processes, including angiogenesis, apoptosis, cell cycle, and proliferation, along with tumorigenic functions, including cell migration, invasion, and metastasis [22].

Despite inhibiting the growth and viability of different cancer types, TQ has no adverse effects on healthy cells [23]. Health-promoting effects and distinct advantages of TQ are primarily associated with the presence of lipophilic quinine components in its structure. The lipophilic nature of TQ enhances its accessibility to cellular and subcellular structures by targeting intracellular transcription factors and kinases and interfering with oncogenesis [24]. The purpose of this review is to provide a comprehensive report of the in vitro and in vivo investigations of the anticancer characteristics of TQ against osteosarcoma in the literature.

## Osteosarcoma pathogenesis

The development of OS is attributed to various complicated phenomena, including genome instability, chromosomal abnormality, and some specific syndromes. OS originating from cortical surfaces of bones is split into three distinct types: parosteal, periosteal, and high-grade OS [25]. Parosteal osteosarcoma, a subclass of low-grade OS, has a fibroblastic-like appearance and is limited to the surface of bone structures; however, it may gradually spread to interior bone tissues. The only treatment for parosteal osteosarcoma that has been shown to lead to a favorable prognosis is surgery. Periosteal osteosarcoma, as the single intermediate-grade subclass, presents chondroblast histology, and it is generally treated with systematic chemotherapy [10]. High-grade OS, known as the classic osteoblastic subclass, is the most progressive and devastating type. This subclass is regarded as a micrometastatic carcinoma at diagnosis stages and, as mentioned previously, is treated with a combination of chemotherapy and surgery [26]. One of the factors allowing OS cells to proliferate is their resistance to apoptosis. Anoikis is a type of apoptosis that causes cells to detach from their component matrix. OS cells are extremely resilient to anoikis, and they may proliferate despite the attachment of cell–cell and cell–matrix being disrupted [27].

Various syndromes such as Bloom’s syndrome, Li–Fraumeni syndrome, retinoblastoma, Rothmund–Thomson syndrome, and Warner’s syndrome predispose to osteosarcoma. Li–Fraumeni syndrome is the syndrome that shows the highest susceptibility to pediatric sarcoma [28]. *TP53* gene, which encodes p53, undergoes a germline mutation in Li–Fraumeni syndrome. p53 is a transcription factor modulating gene-associated DNA repair and triggering post-damage apoptosis [29, 30]. Evidence shows that approximately 30% of individuals with Li–Fraumeni syndrome develop OS. Moreover, 18–26.5% of sporadic osteosarcoma cases lack somatic p53 [31, 32]. Retinoblastoma is another syndrome that may lead to OS. The *RB1* gene binds to the E2F transcription factor family and encodes the pRb retinoblastoma protein. Generally, lack of pRb arises in OS sporadic cases and results in unfavorable outcomes [33]. The incidence of OS is higher in patients suffering from various infrequent autosomal recessive diseases, including Bloom’s syndrome, Rothmund–Thomson syndrome, and Warner’s syndrome. Such syndromes are consequences of RecQ helicase genes [34].

One of the indicators of OS is high expression of midkine suppressing apoptosis processes and enhancing OS cell proliferation [35]. The extent of OS, including its persistent growth as well as its metastasis to other tissues such as bone and lung, is highly dependent on tumor angiogenesis [36]. In OS, the levels of antiangiogenic proteins such as troponin I and pigment epithelial-derived factor (PEDF) reduced, while those of several growth and angiogenic factors, including interleukin 8 (IL-8), vascular endothelial growth factor (VEGF), epithelial growth factor receptor (EGFR), and platelet-derived growth factor receptors (PDGF-R), are increased. Furthermore, in metastatic OS, particular genetic alterations occur, including upregulation of Notch1 and Notch2 receptors along with proto-oncogene tyrosine-protein kinase Src (Src) and wingless-type MMTV integration site family (Wnt)/β-catenin pathways and downregulation of the Fas and Fas ligand (FASL) pathway [37]. Insulin-like growth factor type 1 receptor (IGF-R1) pathway causes expression of mitogen-activated protein kinase (MAPK)/extracellular signal-regulated kinase (ERK) and phosphoinositide 3-kinases (PI3K)/protein kinase B (Akt)/mammalian target of rapamycin (mTOR) to decline, which may eventually lead to enhanced survival, proliferation, and migration of OS cells [38]. Bone and bone marrow tissues have abundant mesenchymal stem cells (MSCs) that are situated close to OA cells. Various in vitro and in vivo observations have revealed that MSCs promote OS cell proliferation [39]. As a type of cysteine protease, cathepsin K (Cat K) is produced by osteoclasts and is capable of degrading osteonectin, osteopontin, and collagen, facilitating the invasion process [40].

Among the environmental parameters that may function as OS carcinogens, ionizing and ultraviolet radiation are acknowledged [41], with radiation exposure accounting for about 2% of OS occurrences. As an interval of 10–20 years is reported between radiation exposure and OS onset, this parameter is not considered in pediatric OS [42]. Additionally, it has been reported that numerous chemical compounds such as aniline dyes, asbestos, beryllium oxide, chromium salts, methylcholanthrene, and zinc beryllium silicate may be related to OS formation [43].

## Anticancer effects of thymoquinone

**Fig. 1:**
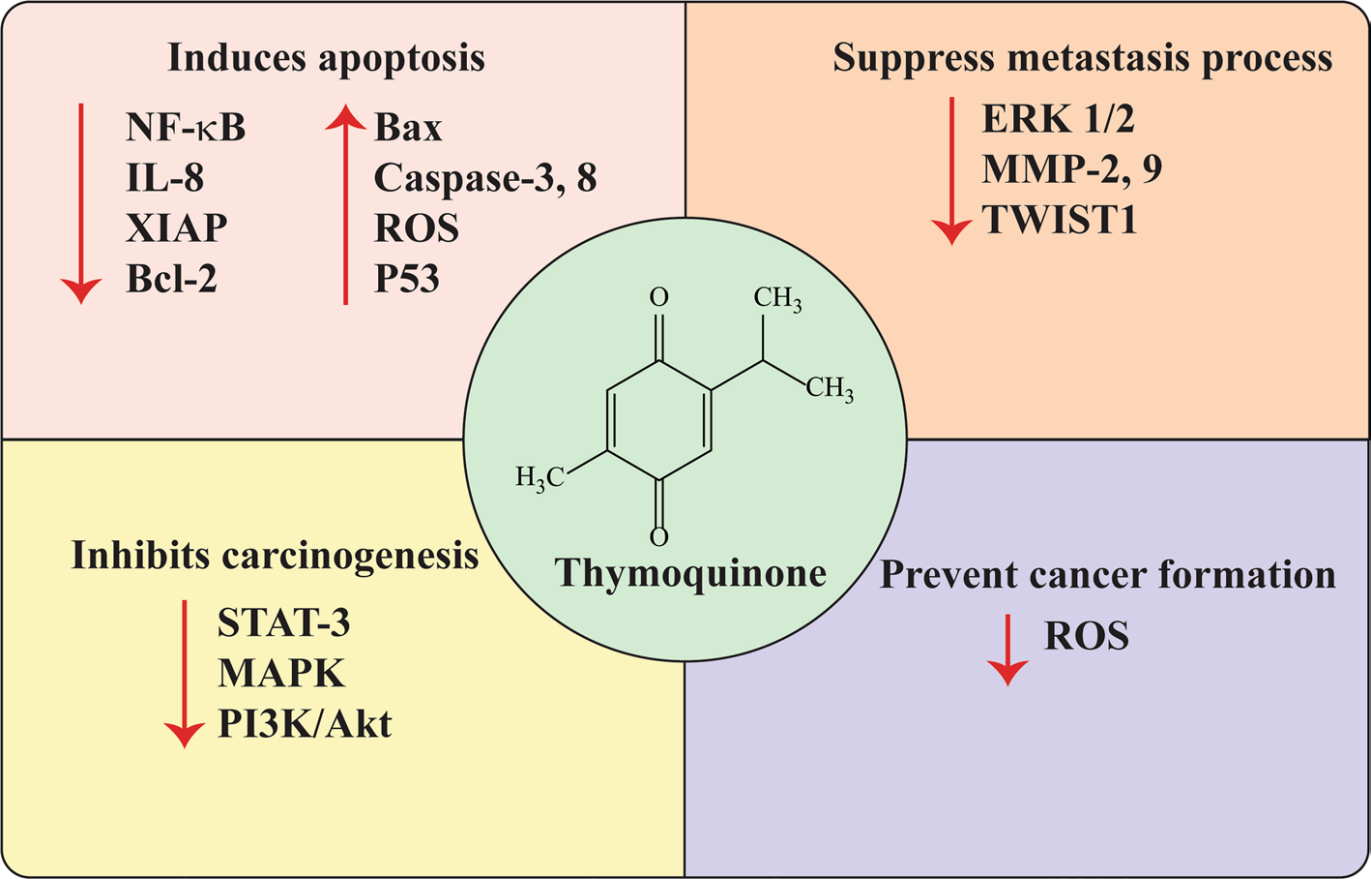
Anticancer properties of thymoquinone

As mentioned previously, TQ has demonstrated profound antineoplastic impact on several types of cancer, including bladder, bone, breast, colon, gastric, lung, prostate, and ovarian, by affecting signaling pathways and/or different cell processes (Fig. 1, Table 1). Based on the report published by Sung et al. (2021), female breast cancer surpassed lung cancer in 2020 and ranks as the most commonly diagnosed cancer, with 2.3 million new cases in 2020 [44]. As the cancer with the fifth-highest mortality rate, it led to 685,000 deaths. Hence, desperate attempts have been made to control breast cancer. In an investigation conducted by Dastjerdi et al. (2016) on the treatment of MCF-7 breast cancer cell lines, p53 was revealed to be one of the targets of TQ [45]. After subjecting MCF-7 cells to a range of TQ concentrations and treatment durations , they indicated that TQ upregulated the expression of p53 in a time-dependent manner, promoting apoptosis in MCF-7 and, consequently, reducing the proliferation of cancer cells. In another study, Khan et al. (2015) found that application of TQ to BT 549 cell lines (breast cancer cells) in a dose-dependent fashion reduced the transcription activity of TWIST1, one of the promotors of endothelial-to-mesenchymal transition (EMT) [46]. Moreover, TQ engagement increased the expression of E-cadherin and decreased the expression of N-cadherin genes associated with TWIST1. As a result, TQ could inhibit cancer cell migration and invasion. Zhou et al. [47] studied the antitumor effect of TQ treatment on p. H1047R and p. H1047L, two hotspot mutations of PIK3CA in metastatic breast cancer (BMC). p. H1047R and p. H1047L mutations reduce the inhibitory effect of ΔNp63a, the main isotype protein of the p53-associated p63 expressed in epithelial cells, on the kinase regions of PIK3CA, which may result in augmented activity of PI3K downstream signals.

**Table 1:** Summary of therapeutic characteristics of TQ in various types of cancer

Cancer type	Cell lines	Animal model	TQ dosage	Mechanism of TQ action	Overall outcome	References
Bladder cancer	T-24 and HTB-9 cell lines	–	In vitro: 10 μM in vivo: –	↑: – ↓: Snail, Slug, N-cadherin, mTOR	Inhibition of EMT process	[130]
	T-24 and 253 J cell lines	–	In vitro: 20–80 μM in vivo: –	↑: Bax, cytochrome C release, caspase-3, caspase-7, caspase-9, GRP78, CHOP, PERK, IRE1, ATF6 ↓: Bcl-2	Apoptosis induction via targeting endoplasmic reticulum stress-dependent mitochondrial pathway	[131]
	T-24 and 253 J cell lines	Xenograft mouse	In vitro: 20–40 μM in vivo: 10 mg/kg/3 days	↑: E-cadherin and ↓: N-cadherin, vimentin, Wnt/β-catenin, MYC, axin-2, MMP7, cyclin D1, and MET	Inhibition of EMT and metastasis processes, decrease in tumor weight	[132]
Breast cancer	BT-474, MCF-7, and MDA-MB-231 cell lines	–	In vitro: 10–30 μM in vivo: –	↑: Caspase-7, caspase-8, caspase-9, PPAR-γ ↓: Bcl-2, Bcl-xL, survivin	Induction of apoptosis, cell cycle arrest, and antiproliferative effects	[133]
	Doxorubicin-resistance MCF-7 cell lines	–	In vitro: 100 μM in vivo: –	↑: Bax, p53, p21, PTEN, caspase-3, caspase-7, caspase-9, PARP cleavage ↓: Akt, Bcl-2, cyclin B1	Apoptosis induction and disruption of mitochondrial membrane potential and cell cycle arrest at the sub-G_1_ phase	[134]
	MDA-MB-468 and T-47D cell lines	–	In vitro: 12.5, 18 μM in vivo: –	↑: Bax, cytoplasmic cytochrome c, p53, p21, procaspase-3, PARP cleavage ↓: Akt, Bcl-2, Bcl-xL cyclin D1, cyclin E, survivin	Cell cycle arrest at the G_1_ phase and apoptosis induction	[135]
	MDA-MB-231 and MDA-MB-468 cell lines	–	In vitro: 2.5, 5 μM in vivo: –	↑: AIF, caspase-3, caspase-8, caspase-9, cytoplasmic cytochrome c, γH2AX ↓: Akt, XIAP, PARP-1	Cell cycle arrest at the G_1_ phase and apoptosis induction in mutant p53 cells	[136]
	MCF-7 and MDA-MB-231 cell lines	–	In vitro: 10–100 μM in vivo: –	↑: – ↓: –	Significant decrease in the viability of cancer cells	[137]
	MDA-MB-231 and MDA-MB-436 cell lines	Xenograft mouse	In vitro: 5–15 μM in vivo: 20, 100 mg/kg/3 day	↑: miR-603 ↓: eEF-2 K, NF-κB	Inhibition of cell proliferation, migration, and invasion, decrease in tumor weight	[138]
	MCF-7 and TD47 cell lines	–	In vitro: 0.01–300 μM in vivo: –	↑: – ↓: –	Augmentation of gemcitabine anticancer activities through upregulation of apoptosis and autophagy processes	[139]
	MDA-MB-231 and MDA-MB-436 cell lines	Xenograft mouse	In vitro: 0–45 μg/ml in vivo: 5 mg/kg/day	↑: miR-361 ↓: Rac, RhoA, VEGF-A	Angiogenesis and metastasis suppression and tumor burden reduction, decrease in tumor weight	[140]
Cervical cancer	SiHa cell lines	–	In vitro: 1–30 μg/ml in vivo: –	↑: p53 ↓: Bcl-2	Cell cycle arrest at the sub-G1 phase, induction of apoptosis and necrosis	[141]
	HeLa cell lines	–	In vitro: 12.5–100 μM/ml in vivo: –	↑: BCL2L10, BIK, caspase-1, FASL ↓: NF-κB	Increase in cell death, promotion of apoptosis	[59]
	CaSki and SiHa cell lines	–	In vitro: 1–40 μM/ml in vivo: –	↑: E-cadherin ↓: TWIST1, Zeb-1	Induction of apoptosis, inhibition of EMT, migration, and invasion processes	[142]
Colorectal cancer	HCT 116wt, DLD-1, HT29 cell lines	–	In vitro: 40 μM in vivo: 25 mg/kg/day	↑: – ↓: ERK1/2, MEK. PAK1	Decreased cell viability, induction of apoptosis and necrosis, decrease in tumor weight	[143]
	Irinotecan (CPT-11)-resistant LoVo cell lines	–	In vitro: 0–8 μM in vivo: –	↑: Atg7, atg12, becline-1, LAMP2, KC3-II, JNK, p38 ↓: IKKα/β, NF-κB, Snail, Twist, vimentin, MMP-2, MMP-9, ERK1/2, PI3K	Increased cell rate, mitochondrial membrane permeability, induction of apoptosis and autophagy	[144]
	Irinotecan (CPT-11)-resistant LoVo cell lines	–	In vitro: 0–10 μM in vivo: –	↑: JNK, p38, ↓: IKKα/β, NF-κB, Snail, Twist, vimentin, MMP-2, MMP-9, ERK1/2, PI3K	Suppression of metastasis and EMT processes	[145]
	5FU-resistant HCT116 cell lines	Xenograft mouse	In vitro: 0–100 μM in vivo: 20 mg/kg/2 days	↑: p21, p53, γH2AX, ↓: CD44, EpCAM, ki67, NF-κB, MEK	Induction of apoptosis and reduced cell invasion and migration, decrease in tumor weight	[146]
Gastric cancer	BGC-823, HGC-27, MGC-803, and SGC-7901 cell lines	Xenograft mouse	In vitro: 25–100 μM in vivo: 20 mg/kg/day	↑: Bax, caspase-3, caspase-9, cytochrome c ↓: Bcl-2	Increased sensitivity to 5-FU, induction of apoptosis, decrease in tumor weight	[147]
	BGC-823, HGC-27, and SGC-7901 cell lines	Xenograft mouse	In vitro: 10–125 μM in vivo: 10–30 mg/kg/2 days	↑: Bax, caspase-3, caspase-7, caspase-9 ↓: Bcl-2, cyclin D, c-Src, JAK2, STAT3, survivin, VEGF	Inhibition of cell growth and angiogenesis, apoptosis induction, and reduction of tumor weight	[50]
	HGC-27, MGC-803, and SGC-7901 cell lines	Xenograft mouse	In vitro: 5–80 μM in vivo: 10 mg/kg/2 days	↑: AIF, Bax, caspase-3, caspase-9, cytochrome c, PTEN ↓: Bcl-2, cyclin D1, p-gp	Increased sensitivity to cisplatin, induction of apoptosis, decrease in tumor weight	[148]
	AGS, SNU638, and SNU719 cell lines	Xenograft mouse	In vitro: 5–50 μM in vivo: 5 mg/kg/2 days	↑: E-cadherin, TTP ↓: MUC-4, N-cadherin, Slug, Snail, TWIST	Reduced cell proliferation, metastasis, EMT process, and tumor weight	[149]
Glioblastoma	CCF-STTG1 and U-87 cell lines	–	In vitro: 10–100 μM in vivo: –	↑: – ↓: ERK, FAK, MMP-2, MMP-9	Reduced cell survival, migration, adhesion, and metastasis processes	[150]
	S6 cell lines		In vitro: 10–100 μM in vivo: –	↑: – ↓: ERK, JNK, NF-κB, p38, PKC	Induction of apoptosis and necrosis, ROS generation, promotion of cell cycle arrest, mitochondrial dysfunction	[151]
Liver cancer	SNNC-7721 and HepG2 cell lines	–	In vitro: 20–100 μM in vivo: –	↑: Bax, caspase-8 ↓: Bcl-2, VEGF	Cell cycle arrest at G_2_/M phase and induction of apoptosis	[152]
	–	Xenograft rats	In vitro: – In vivo: 20 mg/kg/day	↑: Caspase-3, caspase-8, caspase-9, TRAIL/TRAILR2, GSH ↓: Bcl-2, TGF-β1, MDA	Suppressed development of cancer cells via reducing oxidative stress and induction of apoptosis, decreasing tumor weight	[153]
	HCC and HepG2 cell lines	–	In vitro: 30– 70 μM in vivo: –	↑: Caspase-3, cleaved PARP ↓: Bcl-2	Pronounced sensitivity of cancer cells to doxorubicin and cisplatin, ROS generation, and apoptosis induction	[154]
	HepG2, Huh7 cell lines	–	In vitro: 6.25–50 μM	↑: Caspase-3, miR-16, and miR-375 ↓: Bcl-2	Increased cell death, stimulated apoptosis, synergy effect of doxorubicin	[155]
Lung cancer	A549 cell lines	–	In vitro: 5–160 μM in vivo: –	↑: P16 ↓: cyclin D1, ERK1/2, MMP-2, MMP-9, PCNA	Decreased rate of cancer cell proliferation, migration, invasion, and metastasis, cell cycle arrest at the G_0_/G_1_ phase	[156]
	A549 cell lines	–	In vitro: 25–50 μM in vivo: –	↑: Bax, caspase–3, caspase-9, p53 ↓: Bcl-2	Decreased cell viability and induction of apoptosis as well as necrosis	[157]
	A549 cell lines	–	In vitro: 5–80 μM in vivo: –	↑: Bax, caspase-3, p53, PARP ↓: Bcl-2	Depolymerization of microtubule and disruption of mitotic spindle organization, promotion of apoptosis, and decrease in cell viability	[158]
	A549 cell lines	Xenograft mouse	In vitro: 0.5–10.5 μM in vivo: 5 mg/kg/day	↑: Bax, caspase-3, caspase-9, miR-16, miR-375, p53 ↓: Bcl-2	Cell cycle arrest at sub-G_0_/G_1_ phase, triggering of apoptosis, and inhibition of metastasis	[159]
Neuroblastoma	Neuro-2a cell lines	–	In vitro: 10–70 μM in vivo: –	↑: Bax, caspase-3, caspase-9, cleaved PARP, cytochrome c ↓: Bcl-2, XIAP	Reduced cell survival, migration, adhesion, and metastasis processes	[160]
	Neuro-2a cell lines	–	In vitro: 10–70 μM in vivo: –	↑: ↓: MMP-2, MMP-9, NF-κB	Induced apoptosis and suppressed invasion and metastatic processes	[161]
Ovarian cancer	ID8_NGL, NCI/ADR, and OVCAR-3	Xenograft mouse	In vitro: 2.5–50 μM in vivo: 20 mg/kg/2 days	↑: Bax, cleaved PARP ↓: Bcl-2, PCNA	Increased cell death, sensitivity of cancer cells to cisplatin, induced apoptosis	[162]
	SK-OV-3 cell lines	–	In vitro: 10–25 μM in vivo: –	↑: Bax, ↓: Bcl-2	Induced apoptosis, cell cycle arrest at the S phase, and reduced anticancer impact of cisplatin	[163]
Pancreatic cancer	AsPC-1, BxPC-3, and PANC-1 cell lines	Xenograft mouse	In vitro: 10–50 μM in vivo: 50 mg/kg/2 days	↑: Bax, caspase-3, caspase-9, cytosolic cytochrome c ↓: Akt/mTOR, Bcl-2, Bcl-xL mitochondrial cytochrome c, Notch1, p65, PTEN, survivin, XIAP	Reduced cell viability, cell cycle arrest at the G_0_-G_1_ phase, induced apoptosis, and increased sensitivity to gemcitabine	[164]
	AsPC-1, Hs766T, and MiaPaCa-2 cell lines	Xenograft mouse	In vitro: 10–50 μM in vivo: 5–30 mg/kg/2 day	↑: Bax, p21, p53 ↓: Bcl-2	Reduced cell survival, cell cycle arrest at the G_0_-G_1_ phase, inhibited histone deacetylation, triggered histone acetylation, induced apoptosis, and decreased tumor size	[56]
	PANC-1 and MiaPaCa-2 cell lines	–	In vitro: 6.25 μM	↑: caspase-3, miR-24–1, miR-101, cleaved-PARP, PKM2 ↓: –	Suppression of cell viability, stimulation of apoptosis, and increased effect of gemcitabine	[165]
Prostate cancer	1–120 μM	DU-145	In vitro: 1–120 μM	↑: ↓: AktPI3K	Decreased cell viability and increased anticancer effect of docetaxel	[166]
	DU-145 and PC-30 cell lines	xenograft mouse	In vitro: 1.25–30 μM in vivo: 5–30 mg/kg/2 days	↑: E-cadherin, ↓: Slug, TGF-β, Smad-2, Smad-3, vimentin	Reduce cell survival, migration, and invasion	[167]
	DU-145, LNCaP, and PC-3 cell lines	–	In vitro: 5–15 μM in vivo: –	↑: – ↓: Akt, IL-7, IL-7R, MMP-3, MMP-7, NF-κB	Inhibition of cell invasion and metastasis	[168]

↑: Increased level

↓: Decreased level

According to global cancer statistics 2020, lung cancer is the second most commonly diagnosed cancer and leading cause of cancer-related death in 2020, with around 2.2 million new cases and 1.8 million deaths. It is the most common cancer-associated morbidity and mortality in men, while in women it is ranked third after breast and colorectal cancer in terms of incidence and has the second-highest mortality rate after breast cancer [44]. Recently, various investigations have been conducted on lung cancer treatment by natural compounds. In a study on the underlying molecular mechanism of TQ on A549 lung cancer cell line, it was revealed that incubation of A549 cells with TQ reduced the expression of proliferating cell nuclear antigen (PCNA) as a proliferation marker, as well as cyclin D1. Additionally, Yang and coworkers found that application of TQ at a dose of 40 μM and at timepoints of 24, 48, and 72 h downregulated cyclin D1, MMP2, MMP9, and PCNA in A549 cell lines. Moreover, TQ, through blocking phosphorylation of ERK1/2, caused proliferation, migration, and invasion of A549 cancer cells [48].

Colorectal cancer has been classified as the second most common cause of cancer mortality and is ranked third in terms of cancer incidence in 2020. Various studies have been carried out on the association of TQ with colorectal cancer. Kundu et al. (2014) examined the effect of TQ on human colon cancer cells (HCT116). They reported that TQ treatment stimulated apoptosis and reduced cancer cell viability in a dose- and time-dependent manner. Investigation of the molecular mechanism underlying TQ antiproliferative effects revealed that TQ upregulated the pro-apoptotic Bax (BCL-2 associated X) protein and downregulated the anti-apoptotic Bcl-2 (B-cell lymphoma-2) and Bcl-xL proteins [49].

Signal transducer and activator of transcription (STAT) signaling pathway consists of a group of proteins that control several signal transducers, including cytokines, growth factors, and hormones, and play a fundamental role in the proliferation and growth of various tumors. TQ treatment has been shown to prevent phosphorylation and nuclear localization in STAT signaling and, consequently, through downregulating the products of its target genes, including c-Myc, cyclin D1, cyclin D2, and survivin, inhibit cell proliferation in colon cancer. Another study, which applied different doses and treatment durations of TQ to human gastric cancer cells, found that TQ reduced phosphorylation of STAT3 and its upstream kinases, including c-Src and Janus kinase-2 (JAK2). Numerous investigations have shown that the MAPK signaling pathway has a substantial effect on the appearance of TQ antineoplastic characteristics. MAPK families perform a crucial function in various complicated cellular processes, including apoptosis, development, differentiation, proliferation, and transformation.

These variations, along with the reduced activity of cyclin D and Bcl-2 and increased expression of caspase-3, caspase-7, and caspase-9, led to cancer cell growth and increased viability [50]. El-Najjar and colleagues (2016) in their experimental work showed that administration of TQ in human colon cancer cells led to the generation of reactive oxygen species (ROS) and reduced the proliferation of cancer cells. Additionally, TQ through phosphorylation of JNK and ERK, caused MAPK to induce apoptosis [51]. Numerous investigations have found the MAPK signaling pathway to have a substantial effect on the appearance of TQ antineoplastic characteristics. Three members of MAPK families have been recognized: classical MAPK or extracellular signal-regulated kinase (ERK), C-Jun N-terminal kinas/stress-activated protein kinase (JNK/SAPK), and p38 kinase [52, 53]. Incubation of pancreatic cancer cells with TQ resulted in reduced mucin 4 (MUC4) expression via the proteasomal pathway and stimulated apoptosis through JNK and p38 kinases. MUC4 is a high-molecular-weight glycoprotein that is irregularly overexpressed in pancreatic cancer cells, and its downregulation is associated with reduced motility and migration of tumor cells [54].

Overexpression of chemokine interleukin-8 (IL-8) is one of the main indicators of hepatocellular carcinoma (HCC), while administration of TQ led to the downregulation of NF-κB signaling in a dose-dependent fashion. TQ treatment also activated caspase-3 and caspase-9, triggering apoptosis, decomposing poly (ADP-ribose) polymerase, and suppressing G_2_/M cell cycle. Moreover, TQ could stunt the growth of HCC cell lines through the generation of ROS, heme oxygenase-1 (HO-1), and aNAD(P)H quinone dehydrogenase-1 (NQO1) as well as inactivation of Bcl-2, IL-8, and their receptors [55]. Another study investigated the effect of TQ treatment on human pancreatic ductal adenocarcinoma (PDAC) through in vitro and in vivo investigations. The results illustrated that TQ could dose-dependently arrest the G_2_ cell cycle and reduce cell growth and viability related to increased expression of p53 and p21 and decreased expression of Bcl-2 and tumor size [56]. Other investigations found TQ to impede the growth of C4-2B and PC-3 prostate cancer cell lines owing to ROS generation. As a consequence, JNK is activated, leading to increased modulation of GADD45α (DNA damage-inducible gene) and AIF (apoptosis-inducing factor-1) and reduced regulation of Bcl-2 associated proteins and, finally, prostate cancer cell death [57]. Additionally, it has been shown that TQ administration resulted in the downregulation of proteins modulated by E2F-1 that are critical for cell cycle progression.

In LNCaP prostate cancerous cells, TQ therapy substantially increased the level of p21^Cip1^ (cyclin-dependent kinase inhibitor 1), p27^Kip1^ (cyclin-dependent kinase inhibitor 1B), and Bax and arrested the G_1_ to S phase transition of cancer cell cycles, along with a dramatic reduction of androgen receptor (AR) and E2F-1-associated proteins, which are required for progression of the cancer cell cycle [58]. Salkar and coworkers (2013) in their investigation on cervical cancer demonstrated that incubation of HeLa cervical cancer cells with TQ (100 μM) induced apoptosis through extending the regulation of pro-apoptotic gens such as BCL2L10, BIK (BCL-2 interacting killer), caspase 1, and FASL while downregulating genes involved in anti-apoptotic roles of NF-κB activity, namely BH3 interacting-domain death agonist (BID), BCL-2 interacting killer (BIK), v-rel avian reticuloendotheliosis viral oncogene homolog A (RELA), v-rel avian reticuloendotheliosis viral oncogene homolog B (RELB), tumor necrosis factor (TNF), TNF receptor superfamily member 10A (TNFRSF 10A), TNF receptor superfamily member 10B (TNFRSF 10B), and TNF receptor-associated factor 3 (TRAF) [59].

However, poor bioavailability, high hydrophobicity (logP = 2.41), low water solubility (0.5 mg/ml in ethanol), high plasma binding, slow absorption, and short half-life, along with the rapid elimination in physiological conditions, are biological barriers of the therapeutic application of TQ. Accordingly, different nano-drug delivery systems have been developed to overcome these barriers [60]. Soni et al. (2015) loaded paclitaxel (PTX) and TQ into poly(d,l-lactide-coglycolide) (PLGA) nanoparticles. The formulated nanoparticles exhibited enhanced anticancer effects on breast cancer MCF-7 cell lines with decreased PTX toxic effect compared with free drugs [61]. El-Ashmawy et al. (2017) encapsulated doxorubicin (DOX) and TQ into F2 gel (fully acetylated poly-*N*-acetyl glucosamine nanofiber). In vitro investigation demonstrated that treatment of mice bearing solid Ehrlich carcinoma with DOX-TQ led to a significant decrease in tumor volume because of Bcl2 downregulation and p53 upregulation compared with free DOX therapies, implying an improvement in the drug delivery and anticancer effects of DOX with reduced cardiotoxicity [62]. Kommineni et al. (2018) reported co-loading of TQ and cabazitaxel (CBZ) in lipospheres allowed the design of efficient delivery systems demonstrating a synergistic effect on breast cancer cell lines. Analysis of the cell cycle and the apoptosis process indicated that TQ–CBZ delivery systems augmented sub-G1 phase arrest, and also cell death due to apoptosis [63]. In another study, Ramzy et al. (2020) utilized TQ-loaded polymeric nanocapsules with 90.5% encapsulation efficiency to target anis amide (AA) in order to target sigma receptors generally overexpressed in colon cancer. The results showed that AA-functionalized TQ nanocapsules had higher cytotoxic effects than nonfunctionalized ones as well as free TQ against colon cancer HT-29 cell lines [64]. Zafar and coworkers (2020) examined low-molecular-weight chitosan (CS)-grafted lipid nanocapsules (LNP) for co-delivery of docetaxel (DTX) and TQ against two drug-resistance breast cancer cell lines, MCF-7 and MDA-MB-231. The results revealed that functionalization of TQ-loaded LNCs with CS enhanced the uptake and endosomal release of TQ and also increased cytotoxicity against MCF-7 and MDA-MB-231 cell lines [65]. Another study showed that co-encapsulation of TQ and DTX in solid lipid nanoparticles fabricated with 1,2-disteraryol-sn-glycerol-3-phosphoethanolamine-*N*-methoxy-poly(ethylene glycol 2000) (DSPE-mPEG) as a shell and D-α-tochopheryl polyethylene glycol 1000 succinate (TPGS) as surfactant remarkably increased the sensitivity of both MCF-7 and MDA-MB-231 cell lines to DTX and intensified antimetastatic effects, preventing cancer cells from migrating. Moreover, in vivo studies in mice bearing Ehrlich ascites carcinoma (EAC) showed that administration of TQ-DTX-DSPE-mPEG-TPGS lipid nanoparticles significantly reduced the oxidative stress and the DTX-related toxicities in liver and kidney tissues [66]. Alaaeldin et al. (2021) encapsulated TQ into spanlastics fabricated from Span 60 and different edge activators. In vitro studies of breast cancer cell lines illustrated that TQ-loaded spanlastics had 11.5-fold more cytotoxic efficiency against MCF-7 compared with free TQ [67]. Therefore, the incorporation of TQ in nano delivery systems can enhance the efficiency of traditional anticancer drugs and alleviate their side effects.

**Fig. 2:**
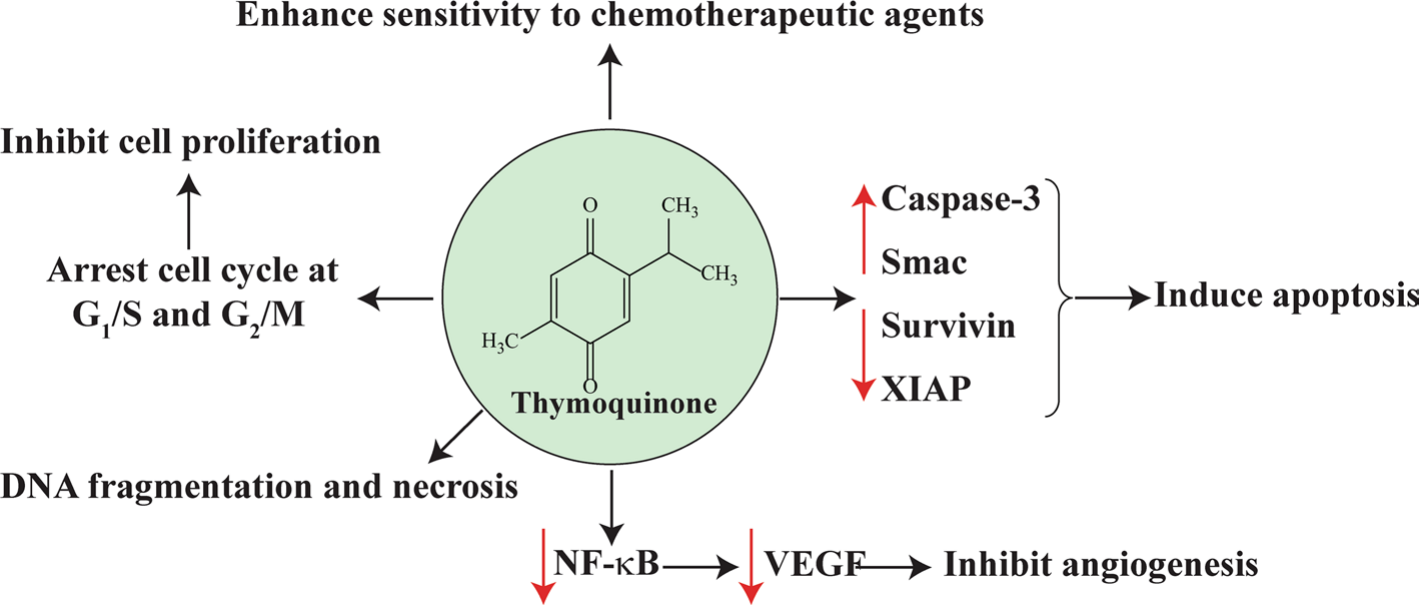
The role of thymoquinone in inhibition of osteosarcoma development

Despite a the small number of in vitro and in vivo studies on the impact of TQ on OS cell lines, therapeutic effects of TQ in this type of cancer are considerable (Fig. 2), and we present a brief review of such observations in the following sections.

## Thymoquinone and osteosarcoma

### Thymoquinone targets signaling pathways

Chronic inflammation and its related disorders are responsible for about 20% of cancer-related deaths. Nuclear factor kappa-light-chain-enhancer of activated B cells (NF-κB) is considered as a class of inducible transcription factors modulating a wide range of genes implicated in various procedures of immune and inflammatory reactions [68]. Under normal physiological conditions, NF-κB is isolated in the cytoplasm; however, it is translocated to the nucleus as a consequence of activating specific signals and then is involved in the transcription of genes managing different cell functions such as cell survival cascades, pro- and anti-inflammatory responses [69], and different types of immune response, such as against bacterial or viral infections [70].

Multiple examinations have shown the swift-active NF-κB to be constitutively involved in osteosarcoma. Conclusive evidence has revealed that the potential of NF-κB signaling to bind to DNA leads to the expression of survivin and X-linked inhibitor of apoptosis (XIAP) and, eventually, induction of apoptosis in different cancer cell types, indicating the role of activated NF-κB in moderating chemoresistant compounds [71, 72]. Conventional chemotherapeutic drugs stimulate NF-κB, resulting in adverse clinical results. Moreover, various NF-κB-associated genes generate products such as VEGF and TNF modulating tumor angiogenesis [73]. Thus, the role of NF-κB is of paramount importance in cancer development, and impeding its function may reduce the rate of angiogenesis and chemoresistant processes; as such, it can be considered as a therapeutic agent against OS. Accordingly, Peng et al. (2013) [74] showed that TQ could abolish the expression of NF-κB in OS cell lines, i.e., SaOS-2. SaOS-2 cells were incubated with different doses of TQ (20, 40, and 80 µM) for 24 h [74]. The outcomes demonstrated that TQ in a dose-dependent manner reduced the rate of DNA-binding activity of NF-κB in SaOS-2 cell lines. Additionally, immunohistochemistry revealed that the expression of the NF-κB protein was considerably attenuated in OS tumors derived from xenograft mouse incubated with TQ (6 mg/kg/day) compared with untreated mouse, indicating the efficiency of TQ administration both in vivo and in vitro [74].

P53 is a signaling pathway well recognized as a “genome guardian” owing to its predominant roles in managing cell processes such as apoptosis, cell proliferation, cell survival, and cell death [75]. The tumor suppressor function of p53 is associated with its capability to induce cell death or reduce cell proliferation. p53 is categorized as a class of transcription factors that either activate or suppress the expression of several genes and miRNAs [76]. Furthermore, p53 has the ability to directly bind to cytoplasmic proteins such as metabolic enzymes as well as apoptotic factors [77]. Additionally, p53 is involved in the response of cells to different types of stress, including DNA damage, hypoxia, oncogenic activation, nutrient variations, etc., through reinforcing cell survival or inducing cell death processes [78]. Reportedly, the p53 mutation has been observed in around 50% of human cancer cases, and discovering a way to preserve it may aid in the prevention of cancer development.

Roepke et al. (2007) investigated the effect of TQ on two human OS cell lines with different mutations of p53, namely MG63 and MNNG/HOS [79]. MG63 cell lines suffered from lack of p53 gene (−/−) as a consequence of a deficiency mutation due to the variation between the first and second exon, while MNNG/HOS cell lines had undergone a point mutation in the codon 156 (CGC to CCC, Arg to Pro) of the p53 gene (±) [80]. TQ promoted p53-independent apoptosis in MG63 cells provided that MNNG/HOS cell lines resisted TQ-associated apoptosis, which might be connected with the capability of these cells to repair DNA damage. This study indicated that TQ administration led to the accumulation of endogenous ROS and DNA damage, including DNA double-strand break (DSB) or base alteration. After DSB damage, phosphorylation of histone H2AX (H2A histone family member X) at the C-terminal residue of serine occurred and produced γ-H2AX, which in turn participates in employing other elements of DNA repair, such as BRCA1, NBS1/Rad50, and p53BP1, to the damaged points. *NBS1*, which is the outcome of mutated genes in Nijmegen breakage syndrome (NBS), acts as an effector of H2AX in response-related DNA damage. The dramatic increase in γ-H2AX in MNNG/HOS cells undergoing p53 mutation without any specific alterations in the expressed levels of H2AX suggests that a novel synthesized species of H2AX is formed following DNA damage. Additionally, the production of γ-H2AX indicates that the DNA damage sensor sensitized to the activity of p53 was functionally intact. The increase in H2AX occurred with anticipated time retardation, and its concentration was reduced at higher TQ dosages. Roepke and coworkers showed that exposure of the p53 null MG63 cells to TQ caused an unchanged and time-dependent reduction in the levels of NBS1 and γ-H2AX, respectively, indicating inadequate DNA repair. Meanwhile, the continuously increased expression of both NBS1 and H2AX in MNNG/HOS cells is compatible with DNA repair being initiated. It is conceivable that mutant p53 is able to repair DNA damage in MNNG/HOS cells, rather than MG63 cells, indicating its role in apoptosis in damaged states. In other respects, H2AX and NBS1 are not particularly crucial for p53 interactions with DSB-related DNA damage and may act in another pathway of p53 [79].

### The effects of thymoquinone on cell proliferation/cycle

**Fig. 3:**
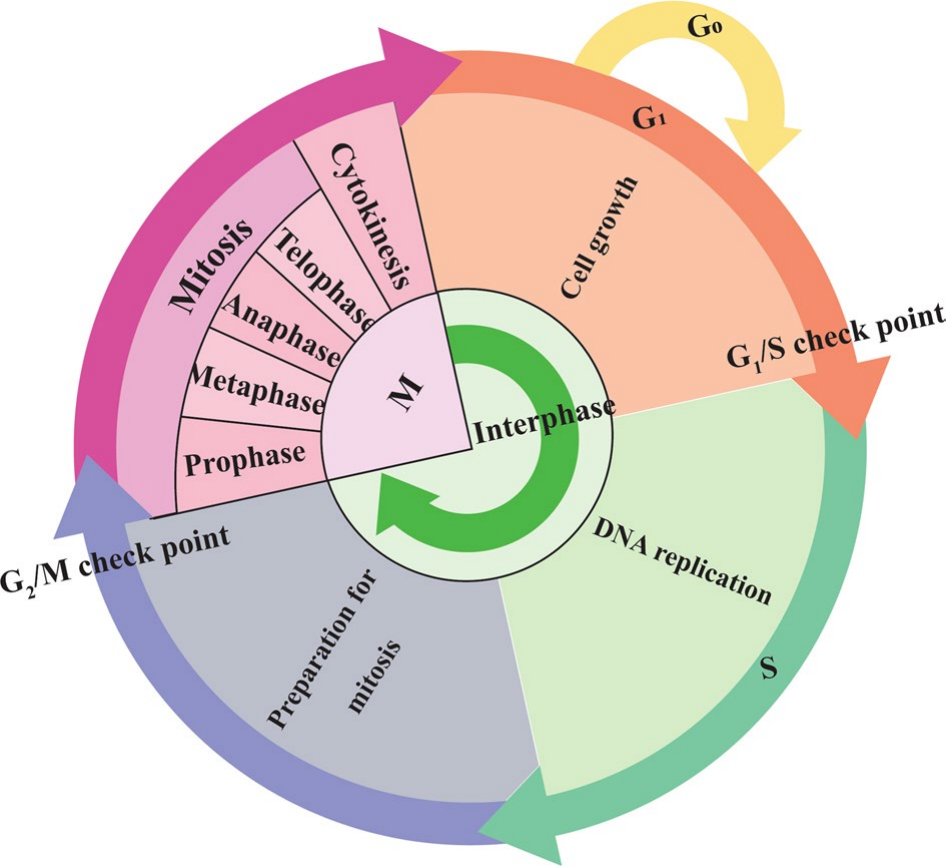
Different phases of the cell cycle and corresponding checkpoints

Cell proliferation refers to the increase in cell number due to cell division, known to be an extremely intricate, strictly managed, and thoroughly regulated process [81]. In normal conditions, the establishment of a proper balance between cell growth and death is mandatory for the normal function of cells, and dysregulation of cell divisions and shifting of the balance to the cell production and increased cell proliferation may lead to cancer [82]. In the highly elaborate process of the cell cycle, a mother cell produces two daughter cells. Thus, controlling the cell cycle and proliferation is one of the suggested ways of inhibiting cancer development [83]. Different phases of the cell cycle are presented in Fig. 3. Briefly, most cells in the mature animals are found in the stable state and at the G_0_ (gap) phase of the cell cycle. When dividing, cells can launch the G_1_ phase. In the majority of cells, DNA replication occurs within a narrow part of the cell cycle known as the S (synthesis) phase. Following the S phase, the cell initiates the second gape phase known as G_2_. In the next steps, or at the M (mitosis) phase, the contents of the nucleus are condensed to organize visible chromosomes and split into two identical collections via a complex regulated stream of movement. Eventually, the mother cell divides into two daughter cells [84]. Cells display an inhibitory effect on the growth of other cells, defined as social control of cell division and regulated through a series of genes known as social control genes.

DNA mutations in a cell cause disruption of the social restraint, and as a result, cells divide without considering the requirements of the organism, which may lead to the development of tumor cells [81]. Mutant genes, if not repaired by DNA repair systems, interfere with the modulation of cell division. Accordingly, the mutant cells that are deprived of a decelerator for cell growth constantly divide, progress, and ultimately transform into malignant cells [85]. Therefore, targeting the cell cycle regulation is one of the therapeutic approaches to cancer treatment. Several studies have revealed that TQ, through suppressing cancer cell proliferation, inhibits cancer development [86].

Roepke et al. (2007) observed the effect of TQ on OS cell line to be highly dependent on the state of p53. Reportedly, after treatment of OS cells with TQ (40 μM for 48 h), the cell viability was about 40% and 80% for MG63 cells (null p53) and MNNG/HOS (mutant p53) respectively. Furthermore, they illustrated that incubation of OS cell lines with 40 μM increased the population of cells in the pre-G_1_ phase in a time-dependent fashion in such a way that, after 48 h, the cell number of MG63 and MNNG/HOS cell lines grew by about 63% and 31%, respectively [79]. The dramatic increase of MG63 cell population in the pre-G_1_ phase was in accordance with the reduction in S and G_2_/M, while TQ after 48 h could arrest MNNG/HOS cell cycle at the G_2_/M phase, along with upregulated expression of p21^WAF1^ protein.

Shoeib and colleagues (2003) report that TQ administration inhibited the proliferation of canine OS cell lines (COS31) in a dose-dependent manner. The result of this examination indicated that, firstly, cell proliferation was predominantly prevented by apoptosis processes and, secondly, the necrosis machinery emerged after a longer duration of treatment as a consequence of lack of white cell phagocytosis in in vitro conditions [87]. Moreover, TQ (100 μM) decreased the population of COS31 cells in the S phase and increased it in the G_1_ phase. Authors concluded that, following TQ incubation, the G1-phase checkpoint was activated, and subsequently, cells might progress through either cell cycle or apoptosis. Therefore, TQ, by targeting two principal processes, namely cell cycle and apoptosis, exerted its inhibitory effect on canine SaOS-2 cells. TQ as an edible quinone compound, through generating free radicals, mediated several alterations in DNA, including alkylating, cross-linking, and double-strand breaking [87]. In another study conducted by Peng and co-workers (2013), the viability of SaOS-2 cell lines decreased dose-dependently (20, 40, and 80 μM) in the presence of TQ after 24 h. Moreover, morphological observations showed that TQ treatment caused the occurrence irregular, condensed, and huge nucleus as well as DNA breakage in SaOS-2 cells.

### The effect of thymoquinone on cell death and induction of apoptosis

**Fig. 4:**
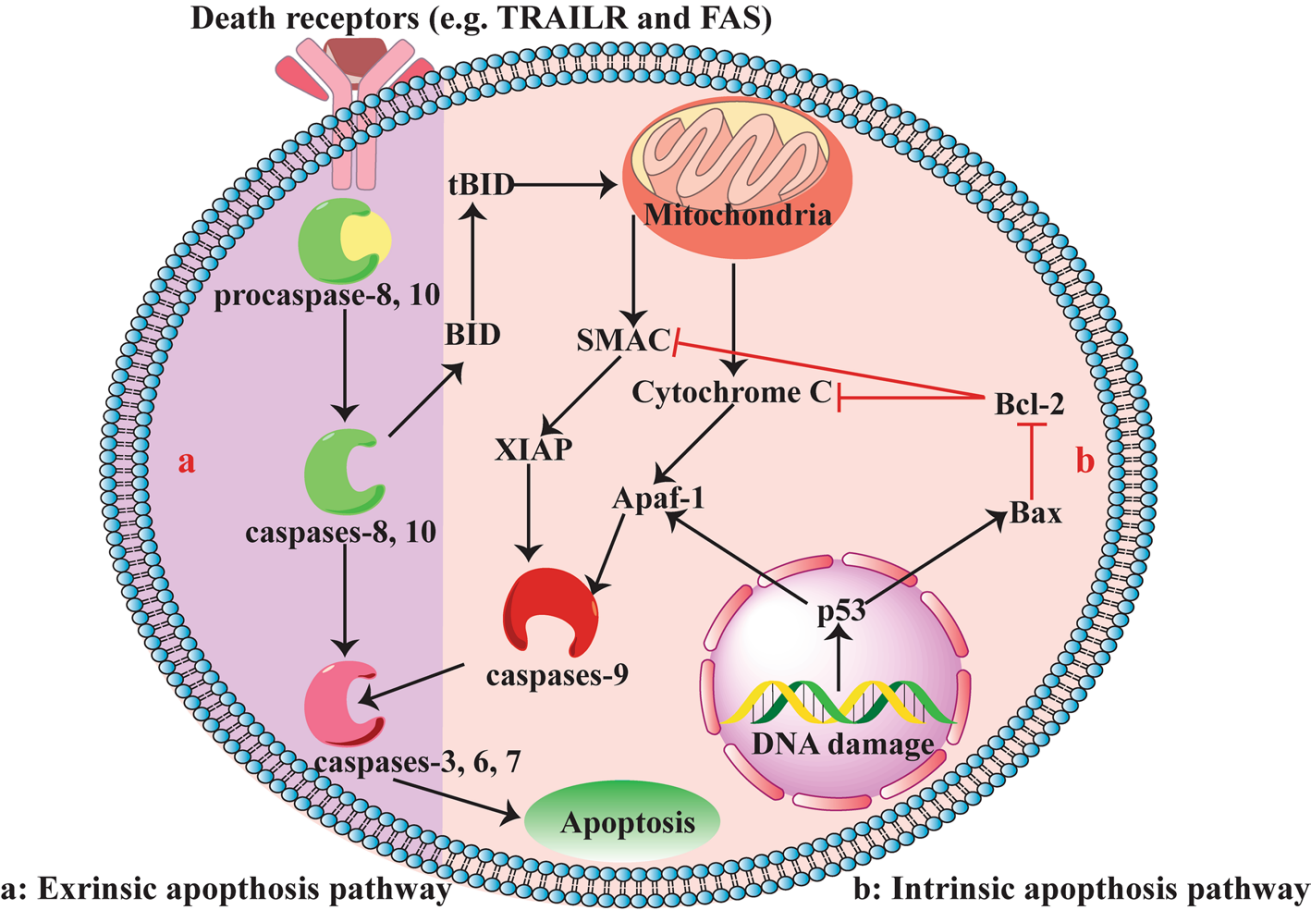
The molecular mechanisms underlying intrinsic and extrinsic apoptosis pathways

Apoptosis, defined as programmed cell death, is an intrinsic mechanism of cells that specifically perform a crucial function in the development and hemostasis of long-lived mammals [88]. As a highly elaborate and modulated process, apoptosis eradicates undesirable and dispensable cells. Numerous conditions cause the signaling of apoptotic pathways, the most important of which are unrestrained proliferation and DNA damage [89]. Apoptosis processes are triggered through either receptor-mediated extrinsic or mitochondrial-mediated intrinsic cascades capable of activating the upstream and downstream caspases (cysteine aspartyl-specific proteases) (Fig. 4) [90, 91]. Caspases are activated immediately after stimulating apoptosis and destroy important cellular components such as nuclear and cytoskeletal proteins required for the normal functioning of cells [88]. Initiator caspases, including caspase-2, -8, -9, and -10, are activated by cellular damage, while executioner caspases, namely caspase-1, -3, -4, -5, -6, and 7, are activated by initiator caspases [89]. In the extrinsic apoptosis pathway or the death receptor pathway, cleavage of particular proteins by executioner caspases leads to DNA fragmentation, nuclear protein damage, protein cross-linking, and ultimately cell death, while the intrinsic or mitochondrial apoptosis pathway is modulated through protein-related BCL-2 family consisting of pro-apoptotic downstream and BH3-only proteins as well as anti-apoptotic BCL-2 proteins [92]. The latter blocks the apoptosis process by suppressing the pro-apoptotic BCL-2 proteins, BAX, and BCL-2 homologous antagonist killer (BAK), while BH3-only proteins block the anti-apoptotic BCL-2 proteins [90] (Fig. 4). It is widely accepted that apoptosis is the prominent mechanism suppressing tumor cells. Thus, the anticancer properties of natural products such as TQ are highly dependent on their apoptotic induction abilities.

Roepke and colleagues (2007) report that TQ (20 μM, 24 h) prompted apoptosis in p53 null MG63 cells, whereas fewer disrupted cells were identified in MNNG/HOS cells. Additionally, it was shown that the number of cytoplasmic histone-related DNA fragments in MG63 cells increased about twofold, while no considerable increment in this index was observed for MNNG/HOS cells after 48 h treatment with 40 μM TQ. This implies that TQ may trigger p53-independent apoptosis in OS cells by stimulating the intrinsic apoptosis pathways [79].

Roepke et al. (2007) employed an immunocytochemical M30 assay to determine the engagement of the mitochondrial pathway in apoptotic effects of TQ in OS cell lines. Cytokeratin, specifically cytokeratin 18, is an intermediate filament protein that is cleaved by caspase-3 or -7 in the early stages of the apoptosis process. M30 CytoDeath is an antibody that is capable of recognizing the particular caspase cleavage position within cytokeratin 18. In p53 null MG63 cell lines, the number of M30-positive cells (caspase cleaved cytokeratin 18) increased about threefold and tenfold after treatment with 20 μM and 40 μM TQ, respectively, while the number of M30-positive cells was not significant in MNNG/HOS cell lines. These results indicated that caspases are involved in the apoptotic effects of TQ.

In another series of examinations, it was revealed that TQ administration stimulated cleavage of initiator caspase-9, which in turn selectively cleaved procaspase-3 in MG63 cells. Further investigations revealed that proteolytic cleavage and activation of procaspase-3 by TQ led to the generation of caspase-3 in MG63 cells.

Bax/Bcl-2 ratio is a crucial index determining the threshold of cells to resist apoptosis. In the presence of a pro-apoptotic compound, Bax is oligomerized on the outer membrane of mitochondria, resulting in increased permeability of the mitochondria to release cytochrome complex (cytochrome c), which induces apoptosis of effector targets such as caspase-9 [93]. Roepke et al. (2007) declared that TQ treatment (40 μM) increased the Bax/Bcl-2 ratio about threefold in both p53 null MG63 and p53 mutant MNNG/HOS cells; however, no significant difference between Bax/Bcl-2 ratio after 24 h or 48 h of TQ treatment for both cell lines indicated that differential apoptosis modulated by TQ was not exclusively due to the regulation of pro-apoptotic Bax and anti-apoptotic Bcl-2 proteins [79]. Moreover, it was observed that increased Bax/Bcl-2 ratio as a consequence of TQ treatment in OS cell lines was due to significant downregulation of Bcl-2. The intensive apoptotic reaction in p53 null MG63 cells may be the secondary effect of a failure to stimulate p53/p21^WAF1^-associated cell cycle arrest.

P21, or p21^WAF1^, is a small protein from the CDK interacting protein/kinase inhibitory protein (CIP/KIP) family of cyclin-dependent kinase (CDK) inhibitors. P21 is an inhibitor of the cell cycle capable of arresting the cell cycle in G1/S and G2/M transition phases by suppressing CDK4,6/cyclin D and CDK-2/cyclin E, respectively [94]. However, various studies have indicated that p21 plays a critical role in carcinogenesis and cancer development through inhibiting apoptosis. Furthermore, p21 inhibits CDKs and increases the expression of genes involved in cell cycle development, DNA repair, and apoptosis regulation, such as E2f family, NF-kB, c-myc, and STAT, resulting in dysfunction of the apoptosis process [95].

Recent investigations have indicated that p21^WAF1^ works in synergy with Bcl-2 to inhibit apoptosis in human lung cancer [96]. The reduced concentration of both Bcl-2 and p21^WAF1^ proteins in p53 null MG63 cells after TQ treatment may cause checkpoint failure and consequently induction of apoptosis in response to DNA damage. However, downregulation of Bcl-2 and slight upregulation of p21^WAF1^ were reported in p53 mutant MNNG/HOS cells. To investigate whether p21^WAF1^ upregulation in MNNG/HOS cells is associated with p53, the small interfering RNA (siRNA) transduction approach, which is a method to knock down a specific gene, was applied. The results of this method indicated that the upregulation of p21^WAF1^ in MNNG/HOS cells was a p53-dependent phenomenon since cells treated with p53 siRNA did not show any increased levels of p21^WAF1^ after TQ administration. It seems that mutant p53 proteins in MNNG/HOS cells are partially active and their transcriptional functions cause the induction of their target gene, i.e., p21^WAF1^.

Therefore, the resistance of p53 mutant MNNG/HOS cells to TQ-stimulated apoptosis may be associated with the capability of these cells to arrest at the G_2_/M phase and repair DNA damage [79].

The potential mechanism inducing apoptosis in both p53 null MG63 and p53 mutant MNNG/HOS cells may be the generation of ROS. Roepke and co-workers (2007) revealed that TQ dose-dependently functioned as a strong promoter to generate mitochondrial O_2_^•−^. The increased levels of γ-H2AX in MNNG/HOS indicate that TQ induces a type of mitochondrial-related apoptosis in these cell lines through increasing oxidative stress [79]. TQ, due to its hydrophobic nature, enjoys high solubility in the lipid part of the inner membrane of mitochondria; moreover, TQ is capable of establishing a redox couple comprising oxidized, semi-reduced, and reduced species of TQ. Accordingly, TQ can integrate into the inner membrane of mitochondria and function as ubiquinone, an electron carrier in the respiratory chain of mitochondria. Thus, oxidized TQ is simply reduced via complex I of the respiratory chain. Additionally, semi-reduced TQ improves the generation of O_2_^•−^ through electron leak from the complex III-associated respiratory chain. Despite mainly operating as a reductant compound, O_2_^•−^ leads to oxidative damage in proteins such as aconitase that possess [Fe-S] cluster in their catalytic domain, causing these proteins to lose their enzymatic activity [97, 98].

In another study, Peng et al. (2013) showed the pro-apoptotic effect of TQ on SaOS-2 cells in a concentration-dependent manner (0, 20, 40, and 80 μM) through upregulation of Smac and caspase-3 as well as downregulation of survivin and XIAP. Furthermore, treatment of xenograft mouse with TQ (6 mg/kg/day) reduced the expression of XIAP as well as survivin while increasing the levels of cleaved caspase-3 and Smac pro-apoptotic activity both in vitro and in vivo [74]. Compelling evidence has suggested that members of the inhibitor of apoptosis protein (IAP) family such as XIAP and survivin, inhibit the activity of caspase-3. During apoptosis, the second mitochondria-derived activator of caspase (Smac) is released from mitochondria into the cytosol, blocking inhibitory effects of IAPs on caspase-3 [99]. Released from mitochondria in response to an apoptotic inducer, Smac binds to the IPAs through an amino-terminal Reaper-associated motif, causing IAPs to displace from their caspase-reacting positions and caspase activation [100]. In an investigation on canine OS cell lines (COS31), it was reported that TQ (0, 25, 50, and 100 μM) dose-dependently augmented the rate of apoptotic cells, as indicated by the increased levels of fragmented DNA in treated cells [87].

### The effects of thymoquinone on cancer cell angiogenesis and metastasis

Angiogenesis is a physiological state in which new blood vessels are developed or generated from pre-existing ones; it is considered as an adaptation mechanism exploited by cells with endothelial origin in both in vitro and in vivo conditions [101]. The angiogenesis process is controlled by multiple components, including angiogenin, angiopoietin, fibroblast growth factors (FGFa and FGFb), hepatocyte growth factor (HGF), interleukin-8, transforming growth factors (TGF-α and TGF-β), tumor necrosis factor (TNF-α), and VEGF, the latter being of considerable importance compared with the others. In vitro examinations have revealed that VEGF induces the growth of endothelial cells predominantly driven by arteries, lymph drainage vessels, and veins [102]. Since the angiogenesis process is required for tumor cell growth, migration, and metastasis, recently, various observations have focused on the inhibition of angiogenesis machinery to restrict the growth of cancer cells and a novel approach for tumor-associated therapies [103]. Peng and co-workers (2013) demonstrated that TQ treatment of SaOS-2 cell lines resulted in reduced expression of VEGF, an indicator of angiogenesis, in a dose-dependent manner. Moreover, in vivo studies on the xenograft mouse exhibited that the administration of TQ (6 mg/kg/day) decreased the level of CD34 [74]. CD34 is an antigen found in hematopoietic progenitor as well as endothelial cells. CD34 is mainly applied for identifying the microvascular vessel density (MVD) as a hallmark of the neoangiogenesis rate [104]. The underlying mechanism is attributed to the NF-κB signaling axis. It has been shown that the angiogenesis of cancerous cells is modulated via NF-κB-related gene products such as TNF and VEGF. Therefore, blocking NF-κB signaling leads to the downregulation of angiogenesis promotors, such as VEGF [73].

Despite substantial progress in early-stage diagnosis and therapeutic approaches of different types of cancer, metastasis remains the main cause of cancer mortality and accounts for 90% of cancer-associated death [105]. Malignant transformation and metastasis arise from genomic alterations of cancer cells as well as environmental and architecture variations of both host and target tissue [106]. Furthermore, the metastasis process is targeted by numerous signaling molecules such as chemotactic stimuli, cytokines, extracellular matrix modifications, and growth factor targets. Consequently, cancer development is generally regarded as a sequenced process through which the phase of a cell metamorphoses from a benign state into an invasive and metastatic classification [107]. The bone is the third most prevalent metastasis site for a broad domain of malignant tumors, including breast, colorectal, gynecologic, lung, melanoma, prostate, and thyroid [108]. It has been reported that bone metastasis occurs in around 70% of metastatic breast and prostate cancer. Following the migration of cancer cells to the bone, treatment of patients seldom leads to improvement, and such a process is accompanied with an extensive variety of morbidities, including hypercalcemia, fracture, and severe pain [109].

Among various proposed approaches to combat bone metastasis, the application of phytochemicals such as TQ is a promising strategy since these compounds show less toxicity against normal cells. Shanmugam et al. (2018) reports that TQ via inhibition of the chemokine receptor type 4 (CXCR4) signaling pathway suppressed osteolytic bone metastasis of breast cancer. Accordingly, mice bearing MDA-MB-231-Luc^+^ expressing cells were treated with TQ of different concentrations, 2 or 4 mg/kg/day, via intraperitoneal injection. Bioluminescence images after 4 weeks revealed that the number of transferred malignant cells to the other distant tissues including bone was significantly less than in control mice without any treatment [110]. Upregulation of CXCR4 is correlated with tumor cell viability, growth, migration, and metastasis. Reportedly, overexpression of CXCR4 has been detected in a wide range of cancer, including cervical, colon, gastric, melanoma, ovarian, pancreatic, renal, and hematological malignancies [111].

The interaction between CXCR4 and its specific ligand, i.e., stromal-derived factor-1 (SDF1 or CXCL12), is of considerable importance in the development of invasion and metastasis of different solid tumors, particularly breast cancer [112]. CXCL12 is a type of autocrine/paracrine growth factor for a variety of cancers and is capable of increasing the level of CXCR4 in triple-negative breast cancer (TNBC) cells. Thus, breast cancer cells with high levels of CXCR4 have a marked tendency to migrate to the sites rich in CXCL12, including bone marrow [113]. In vitro examinations carried out by Shanmugam and colleagues (2018) revealed that pretreatment of MDA-MB-231 cell lines with TQ downregulated the expression of CXCR4 in a time- and dose-dependent fashion owing to reduction of transcript numbers rather than proteolytic cleavage of receptors. This study also showed that TQ reduced the expression of NF-κB signaling, which could be a mechanism underlying the adverse effect of TQ on CXCR4, since the promotor of the *CXCR4* gene has numerous sites to bind to NF-κB [110]. Sharifi and co-workers (2020) applied TQ encapsulated in chitosan nanoparticles to evaluate bone metastasis in hepatocellular carcinoma. A metastasis-on-a-chip platform was designed to model and follow the trend of bone metastasis-associated hepatocellular carcinoma (HCC). The bioreactor designed for this purpose was composed of two chambers containing HepG2 cell lines and a bone-mimetic structure consisting of hydroxyapatite. A microporous membrane above the chambers stimulated the barrier function of vessels when the medium was passed through the membrane. HepG2 cell lines grew in the tumor microtissue, distributed to the circulation flow, and ultimately penetrated the bone chamber. The results indicated that nanoparticle-incorporated TQ could induce antimetastatic characteristics in the bone tissue for a longer time than the samples containing free TQ [114].

### The role of thymoquinone in overcoming drug resistance in osteosarcoma cells

Drug resistance continues to be a formidable obstacle in the development of an appropriate approach to the treatment of various cancer types. Drug resistance is the state in which medical drugs lack enough efficiency and potency to produce effective therapeutic responses [115]. Only a few types of cancer, including pediatric tumors, certain hematological malignancies, and cancerous germ cells, particularly the ones emerging in the testis, are susceptible to chemotherapeutic agents and responsive to treatment [116]. Nevertheless, the common epithelial groups of cancer diagnosed in adults are rarely treatable in the metastatic stage of cancer [116]. DNA mutation and metabolic variations are fundamental causes of inadequacy and degradation of medicines [117]. Drug resistance is classified into two distinct categories: acquired and intrinsic resistance. Acquired resistance involves a series of steps wherein sensitivity of tumors to particular treatments gradually decreases until signs of inefficiency appear. Intrinsic resistance is present in conditions in which malignant tumors present a lack of sensitivity to anticancer drugs without any initial exposure [118]. The suggested procedures for OS treatment include surgery, high-dose chemotherapy via employing chemotherapeutic agents (such as bleomycin, cisplatin, doxorubicin, etoposide, oxaliplatin, taxol, and 5-fluorouracil), and radiotherapy [119].

Despite being effective, chemotherapy regimens are associated with adverse side effects and relatively high cytotoxicity [120]. Furthermore, about 35–44% of cases of OS demonstrate inherent resistance to chemotherapy [119]. Recently, the application of herbal medicines in combination with traditional chemotherapeutic agents for cancer treatment has attracted a great deal of attention owing to its promising results [121]. TQ is one of the phytochemicals that can noticeably inhibit cancer development in combination with chemo- or radiotherapy [122]. It has been observed that TQ in combination therapy, i.e., co-administration of TQ and chemotherapeutic drugs, has profound cytotoxic effects on tumor cells [24]. Sarman et al. (2016) examined the effect of TQ on MG63 cell lines alone and in combination with 5-fluorouracil (5FU) or oxaliplatin (OXA) [123]. It was illustrated that individual administration of the mentioned chemicals (TQ, 5FU, and OXA) to MG63 cells reduced the cell viability in a dose- and time-dependent fashion. Moreover, MG63 cells were treated with TQ at a concentration of 10 or 20 μM in combination with different doses of 5FU or OXA. The result indicated that combining TQ with 5FU at doses 10 μM and 1 μM, respectively, declined the cell viability by around 28% after 72 h while, after this timepoint, the viability of SaSO-2 cells decreased by about 38% as a result of incubation with TQ (10 μM) and OXA (1 μM). The results were astonishing since the individual application of 5FU and OXA at a dose of 1 μM for MG63 cells showed no efficiency, highlighting the promising chemosensitizing role of TQ. Additionally, the results revealed that treatment of MG63 with TQ at a concentration of 10 μM had some apoptotic effect (1.15%), but the combination of 5FU (5 μM) and OXA (1 μM) with TQ (10 μM) increased the apoptotic induction from 35% and 40.2% to 60.35% and 61.65%, respectively [123].

Shoieb et al. (2003) compared the sensitivity of COS31 and its cisplatin (CDDP)-resistant variant (COS31/rCDDP) cell lines to TQ [87]. Results revealed that IC_50_ of COS31 and COS31/rCDDP was 34.8 and 7.7 μM, respectively, suggesting TQ was four to five times more cytotoxic to COS31/rCDDP than COS31 cells. It has been asserted COS31 cells are seven to eight times more sensitive to the cytotoxic effects of higher doses of cisplatin than COS31/rCDDP cells [124]. It has been claimed the high sensitivity of COS31/rCDDP cells to TQ is associated with their augmented expression of glutathione-*S*-transferases (GSTs) [125]. GSTs are a class of enzymes that possess fundamental functions, including anti-apoptotic responses, anti-, and pro-inflammatory activity, regulation of cell signaling, and detoxification of reactive electrophilic substances such as epoxides, nitroso derivatives, hydroxyamines, etc. [126]. One of the critical roles of GSTs is their catalytic activity, they catalyze the conjugation of various electrophilic compounds as byproducts of xenobiotics or reaction-associated oxidative stress to glutathione (GSH), a principal nonprotein thiol compound synthesized de novo in the cells of mammals. The mentioned interaction results in the eradication of cytotoxic compounds from cells and preservation of significant cell components such as proteins and nucleic acids [127].

The detoxification process elucidates the resistance mechanism of particular cell lines to chemotherapeutic drugs since GSTs are upregulated in such cell lines. Moreover, the sensitivity of resistant cells to TQ may be attributed to the overexpression of GSTs. GSH, known as the substrate of GST conjugation, perform a pivotal function in the bioactivation of specific xenobiotics such as quinone compounds. The interaction between quinones and GSH produces quinol–glutathione conjugates with high biological reactivity promoting DNA single-strand breaks; this interplay is indispensable to the cytotoxic effect of this type of chemical [128, 129]. Upregulation of GSTs in CDDP-resistant cells may lead to TQ bioactivity and, ultimately, increased sensitivity of these cell lines to TQ [87]. These results strongly suggest that employing TQ as an adjuvant therapeutic agent along with chemotherapy leads to the adverse effects of chemotherapeutic drugs being minimized owing to their reduced concentrations, thus elevating the efficacy of treatment.

## Conclusions and future perspectives

Poor diagnostic and therapeutic strategies of osteosarcoma and bone metastasis as the principal bone malignancies have led to low rates of patient survival in the past. Although advancements in surgery and chemotherapy methods in recent years have converted increased the lifespan of patients who suffer from such types of fatal disease, the death rate is still high owing to the development of drug resistance and side effects of chemo- and radiotherapies reducing the life quality of patients. Accordingly, increasing the efficiency of the current methods and decreasing their adverse impacts is an important goal, which may be achieved through naturally derived products such as TQ. Various studies have elucidated that TQ, through mediating different processes regulated by various signaling pathways, exhibits anticancer properties.

TQ inhibits bone malignancies through its anti-inflammatory and antioxidant features as well as modulation of various cell-related types of machinery such as angiogenesis, apoptosis, cell cycle and proliferation, and metastasis. Furthermore, TQ with radio- and chemosensitizing effects can reduce the destructive side effects of traditional chemical drugs such as 5-fluorouracil, oxaliplatin, and cisplatin. However, few studies have been conducted in the field of bone malignancies; hence, further investigations, especially in vivo with xenograft mouse, should be considered to reveal other targeting pathways involved in antitumor attributes of TQ.

As TQ is a phytochemical with hydrophobic nature and, consequently, poor bioavailability and pharmacodynamics, it is recommended that more studies be carried out with the focus on enhancing such quality attributes; one potential method is the encapsulation of TQ in nanomaterials. As previously mentioned, numerous investigations have been conducted on the effects of TQ-loaded nano-carriers, either individually or in combination with conventional chemotherapy agents, on various types of cancer, but not OS. Hence, co-application of TQ and traditional chemotherapy drugs in nano-delivery systems in treatment of OS appears to be a promising approach to suppress cancer development and reduce the side effects of current chemical agents because it has been claimed that designing a co-loading of TQ and chemotherapy agents with nano-carrier functionalized for targeting delivery may lead to reduction of concentration and, therefore, toxicity of chemotherapy agents. Another practical strategy for improving the anticancer efficiency of TQ is the synthesis of TQ derivatives that are more stable against various physical, chemical, and physiological conditions while intensifying the toxicity against cancer cells. Among the few studies in the field of OS and TQ, no study has investigated the effect of TQ derivatives on OS. Thus, fabrication of TQ-relative compound, individually or in combination with other effective compounds, functionalized to controlled release and delivery to OS tissues or bone metastasis sites may be an appropriate therapeutic approach to suppress the development of such malignancies, and may be represent a valuable field of future research.

## Data Availability

Not applicable.

## Abbreviations

DNA: Deoxyribonucleic acid
ROS: Reactive oxygen species
OS: Osteosarcoma
TQ: Thymoquinone
PEDF: Pigment epithelial-derived factor
TGF-β: Transforming growth factor beta 1
IL-8: Interleukin 8
PDGF-R: Platelet-derived growth factor receptor
EGFR: Epithelial growth factor receptor
VEGF: Vascular endothelial growth factor
mTOR: Mammalian target of rapamycin
Src: Proto-oncogene tyrosine-protein kinase Src
Wnt/β-catenin: Wingless-type MMTV integration site family
IGF-R1: Insulin-like growth factor type 1 receptor
MAPK: Mitogen-activated protein kinase
ERK: Extracellular signal-regulated kinase
PI3K: Phosphoinositide 3-kinases
Akt: Protein kinase B
MSC: Mesenchymal stem cell
MUC4: Mucin 4
Cat K: Cathepsin K
EMT: Endothelial-to-mesenchymal transition
JNK: C-Jun N-terminal kinase
SAPK: Stress-activated protein kinase
NF-κB: Nuclear factor kappa-light-chain-enhancer of activated B cells
TWIST1: Twist family BHLH transcription factor 1
PCNA: Proliferating cell nuclear antigen
MMP-2: Matrix metalloproteinase-2
MMP-9: Matrix metalloproteinase-9
BCL-2: B-cell lymphoma-2
BAX: BCL-2 associated X protein
BAK: BCL-2 homologous antagonist killer
DSB: Double strand break
NBS: Nijmegen breakage syndrome
STAT: Signal transducer and activator of transcription
JAK2: Janus kinase-2
AIF: Apoptosis-inducing factor-1
BIK: BCL-2 interacting killer
TNF: Tumor necrosis factor
XIAP: X-linked inhibitor of apoptosis
H2AX: H2A histone family member X
CXCR4: Chemokine receptor type 4
5FU: 5-Fluorouracil
OXA: Oxaliplatin
